# A Technical Framework for High‐Throughput Sequencing‐Based Integration Site Analysis in Retroviral Vector‐Based Cell and Gene Therapies

**DOI:** 10.1111/cpr.70292

**Published:** 2026-11-04

**Authors:** Xueling Wu, Yue Chen, Xu Yang, Ning Wu, Wei Wang, Rui Tian, Wanbo Tang, Wenwen Yao, Zhixing Yang, Chao Liu, Yinghua Huang, Kehua Zhang, Shufang Meng

**Affiliations:** ^1^ National Institutes for Drug Control Beijing China; ^2^ State Key Laboratory of Drug Regulatory Science Beijing China; ^3^ Beijing Key Laboratory of Quality Control and Non‐Clinical Research and Evaluation for Cellular and Gene Therapy Medicinal Products Beijing China; ^4^ Key Laboratory of the Ministry of Health for Research on Quality and Standardization of Biotech Products Beijing China; ^5^ BGI Hemogen Therapeutic Shenzhen China; ^6^ BGI Research Shenzhen China; ^7^ Waker Bioscience Co. Ltd. Shanghai China; ^8^ Generulor Co. Ltd. Zhuhai Guangdong China; ^9^ Huzhou Shenke Biotechnology Co. Ltd. Huzhou Zhejiang China; ^10^ BGI Cell Hangzhou China

Advances in cell and gene therapy (CGT) have expanded treatment options for cancers, rare diseases, neurodegenerative disorders and other conditions that respond poorly to conventional therapies. Retroviral vectors, including gamma‐retroviral vectors (γ‐RVs) and lentiviral vectors (LVs), are commonly used in CGT for stable delivery of therapeutic sequences and long‐term transgene expression. However, their semi‐random integration into the host genome may disrupt endogenous gene regulation, activate proto‐oncogenes and contribute to insertional oncogenesis [1]. Integration site analysis (ISA) is therefore an important component of product characterization and safety monitoring during long‐term follow‐up for integrating vector‐based CGT products.

A series of technical guidelines issued by the Center for Drug Evaluation (CDE) of the National Medical Products Administration have established ISA as an important part of the evaluation of CGT products based on integrating vectors [2, 3, 4, 5, 6, 7]. Specifically, these guidelines call for characterization of integration sites (ISs) and their distribution patterns, integration hotspots, clonal expansion and dominant clones and integrations within or near cancer‐related genes to assess potential insertional mutagenic and oncogenic risks. In addition, long‐term follow‐up guidance recommends continued monitoring of integration‐related risks using validated ISA methods in patients treated with integrating vector‐based CGT products [4].

ISA is predominantly performed by high‐throughput sequencing, using either whole‐genome sequencing (WGS) or, more commonly, targeted sequencing following enrichment of vector‐host junctions by methods such as linear amplification‐mediated PCR (LAM‐PCR) and its derivatives [8]. Differences in experimental workflow and analytical performance, together with the lack of standardized approaches to method evaluation, quality control and data interpretation, limit the comparability of ISA results across laboratories and studies, thereby complicating regulatory assessment. To address these challenges, we propose a life‐cycle‐based technical framework for the design, development, validation, quality control, data analysis and interpretation of high‐throughput sequencing‐based ISA for retroviral vectors.

## 1 Scope

This framework applies to high‐throughput sequencing‐based ISA of integrating retroviral vectors, primarily γ‐RVs and LVs, throughout the life‐cycle of CGT products, from investigational new drug (IND) development through new drug application (NDA) and post‐marketing clinical use. Relevant test materials include CGT products and associated clinical samples. The same principles may also be applicable to other integrating gene delivery systems, such as transposons, with appropriate consideration of differences in integration mechanisms and profiles.

## 2 General Principles

The purpose of ISA depends on the sample and the stage of product development. ISA of the drug product mainly focuses on the characterization of the vector integration profile in support of risk evaluation, product quality studies and process optimization. For clinical samples, serial ISA should be performed for long‐term monitoring of risks, with a focus on the variation in the integration profile, clonality and clonal dynamics.

The ISA method should be managed in accordance with ICH Q14 [9], from its initial design and validation to subsequent changes and updates. The intended use of the ISA method should guide its method design, including the selection of performance characteristics and acceptance criteria. When the analytical procedure or its application is modified, the potential impact on the method performance should be evaluated, followed by additional validation performed where necessary. The method should continue to be refined to keep pace with advances in sequencing technology, CGT product development and regulatory expectations.

## 3 Method Design

Method design begins with the establishment of an analytical target profile (ATP) that defines the intended use, analytical objective, relevant performance characteristics and acceptance criteria. The ATP then guides the design of suitable experimental and bioinformatic procedures.

### 3.1 Establishment of the ATP

An ATP for an ISA method should be built around its intended use. ISA supports the assessment of integration‐related risks of relevant CGT products by characterizing IS distribution, clonality and clonal dynamics. The sample type and application determine whether a qualitative or quantitative method is needed and thereby define the analytical objective. The performance characteristics and acceptance criteria of the method should then be specified accordingly. A detailed ATP for ISA is listed in Table S1.

### 3.2 Methodological Design

Current ISA methods differ in the strategies used for DNA fragmentation, target sequence enrichment and quantification (Table S2). Whether and how vector‐host junction sequences are enriched depends on the intended use and leads to different experimental and bioinformatic workflows. Estimating relative IS abundance requires a quantification strategy. Different strategies vary in performance, experimental and bioinformatic workflows, and dependence on the DNA fragmentation strategy. The selection of different approaches should follow the established ATP while also considering practical constraints.

## 4 Method Development

Following method design, method development should be guided by the ATP and informed by prior knowledge of the analytical procedure. Potential sources of variability should be identified through risk assessment and systematically evaluated to establish appropriate operating ranges and ensure robust performance across the intended conditions of use.

### 4.1 ISA Workflow

The ISA workflow comprises experimental procedures and bioinformatic analysis (Figure 1). Experimental procedures typically include sample preparation, sequencing library preparation, and high‐throughput sequencing. Bioinformatic analysis includes raw‐data quality control and preprocessing, reference‐genome alignment, IS identification and annotation, IS distribution and hotspot analysis, and, where appropriate, clonality analysis. Because variability introduced at any stage can affect the final results, the workflow should be developed and evaluated as an integrated analytical procedure.

**FIGURE 1:**
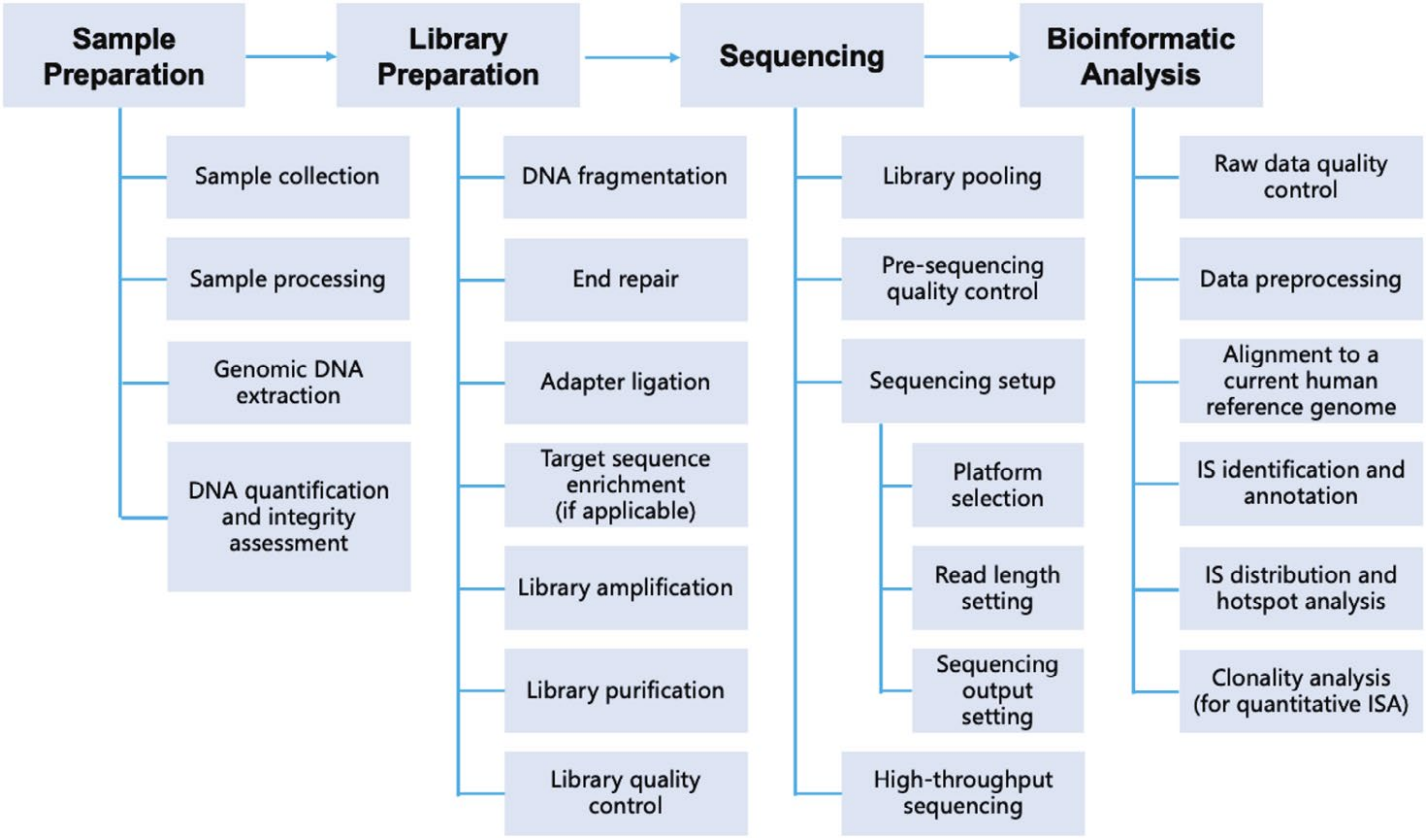
Overview of the integration site analysis workflow.

### 4.2 Evaluation of Key Method Parameters

Factors that may affect analytical performance should be identified and ranked through risk assessment to identify key method parameters. Fishbone diagrams or similar tools may be used to organize sources of variability and identify parameters requiring experimental evaluation.

Appropriate settings and operating ranges for key parameters should then be established experimentally. A one‐factor‐at‐a‐time (OFAT) approach may be suitable for relatively independent variables, whereas design of experiments (DOE) is preferable when interactions among multiple variables are expected. Study design should be aligned with the ATP and provide a scientific basis for parameter control and subsequent method validation.

### 4.3 Key Parameters and Control Strategies

Commonly evaluated key parameters and relevant reference criteria for ISA are summarized in Table S3. Their suitability should be confirmed, and any necessary adjustments should be made and justified for the specific analytical procedure.

Key parameters should be identified and evaluated throughout the ISA workflow, from materials and sample handling to library preparation, sequencing and bioinformatic analysis, with appropriate controls established to support consistent method performance.

General controls should be established for reagents, critical consumables, and samples. Reagents and critical consumables should meet predefined quality specifications and demonstrate acceptable lot‐to‐lot consistency. In‐house‐developed reagents may be used after adequate performance characterization. Applicable sample types should be specified according to the intended use. Procedures for sample collection, transport, storage and freeze–thaw cycles should be specified to limit degradation and cross‐contamination.

The DNA input and library preparation procedures should be optimized for the selected approach. The quantity and integrity of genomic DNA should be evaluated against the predefined criteria before library preparation. When DNA availability is limited for a clinical sample, ISA may still be performed with the actual DNA input recorded, provided that its potential impact on analytical sensitivity and result interpretation is evaluated.

The choice of strategies for DNA fragmentation, target sequence enrichment and IS quantification influences the requirements for adaptor ligation, PCR amplification and other steps, all of which may affect the method performance. Target‐specific primers or capture probes should provide adequate specificity and coverage of the target region. For multiplexed sequencing, barcode combinations should be compatible with the sequencing platform and support accurate sample demultiplexing. For quantitative ISA, the number of independent template molecules may be estimated using unique molecular identifiers (UMIs) or fragment‐end information. UMI design should consider DNA input, library complexity and required analytical sensitivity and its suitability should be demonstrated during method validation.

The sequencing platform and setup should be appropriate for the library structure and required analytical performance. Before sequencing, libraries should be quality‐assessed and pooled appropriately. The required sequencing output should be determined based on the selected ISA methodology, library complexity, required sensitivity and platform capacity. Positive and negative controls should be processed alongside test samples to monitor the assay performance and run‐to‐run consistency.

The companion bioinformatic workflow should be standardized, reproducible and traceable, encompassing raw‐data quality control and preprocessing, sequence alignment, IS identification and annotation, IS distribution and hotspot analysis and clonality analysis, where appropriate. Procedures, parameters, and decision criteria throughout the workflow should be predefined and version‐controlled.

## 5 Method Validation

Following development, the ISA method should be validated in accordance with ICH Q2(R2), Validation of Analytical Procedures [10] and General Chapter 9101, Guidelines for Validation of Analytical Methods, of the Chinese Pharmacopoeia (2025 edition) [11]. The validation protocol and acceptance criteria should be predefined and aligned with the intended use and ATP. Development data may support validation when they adequately address the relevant performance characteristics, avoiding unnecessary repetition.

### 5.1 Preparation and Characterization of Reference Materials

Reference materials used for validation should be well‐characterized, with known ISs and vector copy number (VCN) values. Such materials may be generated by transducing an appropriate host cell line with a retroviral vector and deriving monoclonal cell lines by limiting dilution or single‐cell sorting. ISs may first be identified by ISA and should then be confirmed using at least one orthogonal method, such as Sanger sequencing, WGS, long‐read sequencing, or optical genome mapping (OGM). VCN in the selected clones may be measured by validated droplet digital PCR (ddPCR) or quantitative PCR (qPCR) methods. Together, these methods should confirm the presence and genomic location of each IS.

### 5.2 Validation Characteristics and Acceptance Criteria

Validation should demonstrate that the ISA method meets the performance requirements defined in the ATP. Qualitative ISA generally requires evaluation of specificity, limit of detection and robustness. Quantitative ISA requires assessment of specificity, robustness, accuracy, precision, linearity, range and limit of quantitation. Validation samples should span the intended analytical range and include low‐abundance samples suitable for determining the limit of detection or limit of quantitation, as appropriate. Suggested validation designs and acceptance criteria are provided in Table S4.

## 6 Data Analysis and Interpretation

The scope of data analysis should be defined according to the intended use and ATP. Qualitative ISA supports IS identification, annotation, distribution analysis, and integration hotspot analysis, while quantitative ISA also enables estimation of relative IS abundance and assessment of clonality and clonal dynamics. Results should be analyzed and interpreted only after predefined run‐acceptance criteria have been met and in the context of the sample type and application setting.

### 6.1 Run Acceptance

Each run of ISA should include positive and negative controls and be considered valid only when predefined acceptance criteria are met. The positive control should be set at a low level, approximately two to three times the limit of detection for qualitative ISA or limit of quantitation for quantitative ISA. For the positive control, sequencing data quality and target IS detection should meet predefined criteria and quantitative results should fall within the expected range. The target IS should not be detected in the negative control, with no interfering false‐positive signal. Test samples should also meet the sequencing data quality criteria specified in Table S3.

### 6.2 Scope of Data Analysis

#### 6.2.1 IS Identification and Annotation

IS identification should be based on uniquely mapped reads, using predefined minimum support thresholds and rules for merging nearby sites. A valid IS should generally be supported by at least three reads or independent molecules, as appropriate to the analytical strategy. Adjacent IS calls should be merged using a predefined distance threshold of no more than 20 bp. If reliable IS identification requires a larger merging window, the ISA workflow should be reassessed to identify and address the source of positional uncertainty.

Each IS should be annotated for its genomic context, including the overlapping or nearest gene, its intragenic location where applicable, distance and orientation relative to the nearest transcription start site (TSS) and nearby regulatory elements. For risk assessment, particular attention should be given to ISs located within cancer‐related genes or within ±100 kb of their TSSs. Genes of interest should include proto‐oncogenes and tumour suppressor genes curated in established resources, as well as genes previously implicated in integration‐associated clonal expansion or oncogenesis in gene therapy studies (Table S5). The gene list should be updated in light of new findings.

#### 6.2.2 IS Distribution and Integration Hotspots

The overall integration profile should be characterized across the genome, including chromosomal distribution, GC‐content distribution, distribution across repetitive‐element classes and distribution relative to TSSs and CpG islands. Integration hotspots may be identified using common integration site (CIS) analysis or other suitable statistical or computational methods [12, 13]. Identified hotspots may be further ranked according to the number of independent integration events or the degree of enrichment.

#### 6.2.3 Clonality and Clonal Dominance

Clonality analysis should be based on relative IS abundances generated by a validated quantitative ISA. However, relative IS abundance does not directly represent the proportion of the corresponding cell clone because it reflects the relative contribution of individual integration events and a single clone may harbour more than one IS. Its interpretation should therefore take sample VCN into account. Where sufficient evidence indicates that multiple ISs originate from the same clone, their abundances may be combined for clonal dominance assessment. To further confirm clonal dominance, IS‐specific VCN can be measured by ddPCR, qPCR or another suitable method.

Clonal diversity and the degree of clonal dominance may be assessed using metrics such as the Polyclonal‐Monoclonal Diversity (PMD) index, Pielou's evenness index and Simpson's probability index [14, 15].

High‐abundance ISs and their longitudinal dynamics are central to the assessment of clonal dominance. A dominant clone is characterized by a clear selective growth advantage over other clones and by persistence or expansion during clinical follow‐up. In the absence of a universally accepted numerical definition, the following staged approach may serve as a practical risk‐based framework [16]. For samples with a mean VCN > 0.3 copies per diploid genome, detection of a single IS with a relative abundance > 30%, or of multiple ISs attributable to the same clone with a combined relative abundance > 30%, should prompt repeat ISA within 3 months. If the relative abundance of the corresponding IS(s) subsequently increases to > 50%, IS‐specific VCN should be measured. An IS‐specific VCN > 0.5 copies per diploid genome supports classification of the corresponding clone as dominant.

### 6.3 Risk Evaluation

Risk evaluation should integrate findings from the overall IS distribution, integration hotspots, clonality and gene annotations of relevant ISs. Their significance should be interpreted in the context of sample source, target cell type, vector type, known integration preferences and vector design [17, 18]. Relevant vector design features include self‐inactivating long terminal repeats, the type and activity of internal promoters or enhancers and the presence of insulators or other regulatory elements.

For cell product samples, the overall integration profile and integration hotspots should be compared with previously reported integration patterns for the same vector type [18]. Marked deviations from these typical integration patterns or unusual hotspots should be further evaluated based on the gene annotations of the relevant ISs, with particular attention to cancer‐related genes. Findings from the cell product may also provide a reference for interpreting subsequent clinical follow‐up samples.

Clinical follow‐up samples should be evaluated longitudinally, with attention to persistent or progressive deviations in the overall IS distribution profile, recurrent or increasingly enriched integration hotspots and changes in clonal diversity or the abundance of major ISs. For ISs with high or progressively increasing relative abundance, ISs associated with dominant clones and recurrent or increasingly enriched hotspots, potential relationships among these abnormalities should be examined alongside relevant IS gene annotations. Where appropriate, transcriptomic and other functional studies may be used to investigate whether the observed abnormalities are associated with vector integration, including possible effects on nearby gene expression. For haematopoietic stem and progenitor cell gene therapy (HSPC‐GT), longitudinal ISA of sorted haematopoietic cell lineages may also be performed to characterize clonal expansion patterns and evaluate potential risks. Relevant clinical findings should also be incorporated into the overall assessment, which should then guide subsequent monitoring.

## 7 Conclusions

High‐throughput sequencing‐based ISA is an important component of the life‐cycle evaluation of CGT products using integrating vectors. Reliable ISA depends on a fit‐for‐purpose and well‐controlled experimental and bioinformatic workflow. Furthermore, meaningful risk evaluation requires careful interpretation of IS distribution, integration hotspots, clonality, relevant gene annotations and vector‐related biological context. The framework proposed here provides a structured approach to method design, development, validation, quality control and risk evaluation. As sequencing and analytical technologies continue to evolve, particularly with the increasing use of long‐read and single‐cell approaches, ISA strategies will require continued refinement while upholding the principles of predefined analytical objectives, validated performance, traceable analysis and context‐dependent interpretation.

## Author Contributions

Meng S.F., Zhang K.H. and Liu C. contributed to conception and design. Wu X.L. and Chen Y. drafted and revised the manuscript. Zhang K.H, Meng S.F. Yang X., Wu N., Wang W., Tian R., Tang W.B., Yao W.W., Yang Z.X., Liu C. and Huang Y.H. critically read and revised the manuscript.

## Funding

This work was supported by the National Science and Technology Major Project (2025ZD1803200) and State Key Laboratory of Drug Regulatory Science Project (2025SKLDRS0317).

## Supporting information


**Table S1:** Analytical target profile for integration site analysis.
**Table S2:** Comparison of methodological strategies for integration site analysis.
**Table S3:** Key method parameters and recommended control criteria.
**Table S4:** Validation study designs and acceptance criteria for integration site analysis.
**Table S5:** Genes implicated in integration‐associated clonal expansion or oncogenesis.

## Data Availability

Data sharing not applicable to this article as no datasets were generated or analysed during the current study.
