# Supplementary material for: A Technical Framework for High‐Throughput Sequencing‐Based Integration Site Analysis in Retroviral Vector‐Based Cell and Gene Therapies
Source: Cell Prolif. 2026 Nov 4:e70292. Online ahead of print. doi: 10.1111/cpr.70292 (PMC13732750; doi:10.1111/cpr.70292)
Supplement: Supplementary file 1 [file CPR-9999-e70292-s001.docx]

**Supplementary Table S1. Analytical Target Profile for Integration Site Analysis**

| **Intended use** | To detect and analyse retroviral vector integration sites in CGT products and associated clinical samples for the assessment of integration-related risks. | |
| --- | --- | --- |
| **Relationship to CQAs and analytical objectives** | IS distribution and clonal composition are critical quality attributes (CQAs) relevant to the assessment of potential oncogenic risk associated with vector integration in CGT products. ISA method design should therefore adequately account for the diversity and complexity of integration events, ensure sufficient coverage across genomic regions, and, for quantitative ISA, support reliable assessment of clonal composition. | |
|  | | |
| **Performance characteristics** | **Acceptance criteria** | **Rationale** |
| **Specificity** | All known ISs in the positive reference material should be correctly detected, with no target IS detected in the negative reference material. | Performance characteristics and acceptance criteria should be established based on the Chinese Pharmacopoeia, relevant guidelines, prior knowledge, and the intended use of ISA, to ensure the appropriate quality and reliability of the reported results. |
| **Accuracy** | Recovery should generally be 75%–125% across tested levels. Wider acceptance criteria may be applied at the LOQ where justified. |  |
| **Precision** | Repeatability and intermediate precision should generally have an RSD ≤20% at each tested level; an RSD ≤25% may be acceptable at the LOQ. |  |
| **Linearity and range** | The coefficient of determination (R²) for linear regression should generally be ≥0.95. The analytical range should include levels for which acceptable accuracy, precision, and linearity are demonstrated. |  |
| **Limit of detection (LOD)** | The lowest level at which all known ISs are correctly detected in all replicates. The LOD should generally be ≤0.1% (1 in 1,000 cells). |  |
| **Limit of quantitation (LOQ)** | The lowest level at which predefined accuracy and precision requirements are met. |  |
| **Robustness** | For qualitative ISA, deliberate variations in analytical conditions should not affect correct IS detection. For quantitative ISA, results should continue to meet predefined accuracy and precision criteria. |  |

**Supplementary Table S2. Comparison of Methodological Strategies for Integration Site Analysis**

| **Key methodological aspect** | **Strategy** | **Advantages** | **Limitations** |
| --- | --- | --- | --- |
| **DNA fragmentation** | Enzymatic fragmentation | Simple workflow; high throughput | May introduce sequence-dependent fragmentation bias |
|  | DNA sonication | Relatively random fragmentation; good coverage uniformity | Requires specialized equipment; more complex operation |
| **Target sequence enrichment** | WGS without target enrichment | Simple workflow; enables simultaneous assessment of vector sequence variation | Requires substantially greater sequencing output and generally provides lower sensitivity for IS detection |
|  | Vector-specific PCR enrichment | Relatively simple workflow; efficiently enriches vector-genome junctions | Requires prior knowledge of the vector sequence |
|  | Biotinylated PCR product capture | Efficient enrichment of target sequences | Requires prior knowledge of the target sequence; more complex workflow |
|  | Probe-based hybridization capture | Enables targeted enrichment with reduced dependence on junction-specific PCR amplification | Requires probe design based on known vector sequences; relatively high cost and workflow complexity |
| **Quantification** | No quantification | Simple workflow | Does not provide IS abundance information |
|  | Read-based quantification | Simple and computationally straightforward | Strongly affected by PCR amplification bias and therefore provides limited quantitative accuracy |
|  | Fragment-based quantification | Reduces the influence of PCR amplification bias by estimating the number of independent DNA fragments | Requires sufficiently random fragmentation and additional bioinformatic processing |
|  | UMI-based quantification | Enables molecular deduplication and estimation of the number of independent template molecules | Requires appropriate UMI design and additional experimental and bioinformatic processing |

**Supplementary Table S3. Key Method Parameters and Recommended Control Criteria**

| **Control stage** | **Parameter** | **Recommended criteria** |
| --- | --- | --- |
| **Sample preparation** | **Genomic DNA quality** | DNA concentration and total input should meet the validated requirements for library preparation. DNA integrity should be assessed by agarose gel electrophoresis, capillary electrophoresis, or microfluidic electrophoresis, with no substantial degradation observed. |
| **Library preparation** | **Final library quality** | Library concentration and total yield should meet the requirements of the sequencing platform. The fragment-size distribution should be within the expected range, with no substantial adapter dimers or other abnormal peaks. |
| **Sequencing** | **Raw sequencing data quality** | The percentage of bases with Phred quality scores ≥30 (Q30) should meet the quality performance requirements of the sequencing platform and strategy and should generally be ≥85%. Sequencing output should be sufficient for the intended analysis. |
| **Bioinformatic analysis** | **Raw-data quality control and preprocessing** | Low-quality bases and adapter sequences should be trimmed as appropriate, while reads of insufficient quality or length, or with a high proportion of ambiguous bases, should be filtered out. The retained reads should be of sufficient length and quantity for IS identification and downstream analysis. |
|  | **Sequence alignment** | Reads should be aligned to the latest human reference genome assembly using predefined alignment software and parameters, with uniquely mapped reads retained for IS identification. The reference genome version, alignment software, and parameters should be version-controlled. |
|  | **IS identification** | Predefined IS support thresholds should be applied to exclude low-confidence signals and reduce false-positive calls. A valid IS should generally be supported by ≥3 reads or independent molecules, as appropriate to the analytical strategy. Nearby ISs should be merged using predefined criteria to account for positional variation introduced during PCR amplification and sequencing. The merging distance should not exceed 20 bp. |
|  | **IS annotation** | Database versions should be fixed and traceable. IS annotation should be performed using the same reference genome assembly as sequence alignment and should include relevant gene and genomic-context information. |
|  | **IS distribution analysis** | Analyses should be based on consistently annotated ISs. Analysis dimensions, statistical units, and relevant parameters should be predefined and applied consistently. |
|  | **Integration hotspot analysis** | The method used for hotspot identification and the relevant analysis parameters should be predefined. When CIS or other statistical approaches are used, the analysis method and parameter settings should be consistently applied and adequately documented to ensure reproducibility and traceability. |
|  | **Clonality analysis** | The method used to calculate relative IS abundance should be predefined according to the quantitative strategy, for example using UMI-based or fragment-based quantification. Metrics, calculation methods, and relevant parameters used for clonal diversity analysis should also be predefined and applied consistently. |

**Supplementary Table S4. Validation Study Designs and Acceptance Criteria for Integration Site Analysis**

| **Validation characteristics** | **Qualitative ISA** | **Quantitative ISA** | **Study design** | **Acceptance criteria** |
| --- | --- | --- | --- | --- |
| **Specificity** | + | + | Use a monoclonal cell line with well-characterized vector ISs (or its DNA) as the positive control, preferably one harbouring multiple known ISs. Use the corresponding untransduced cell line (or its DNA) and a monoclonal cell line transduced with a non-target viral vector (or its DNA) as the negative controls. Perform three replicate measurements. | All known ISs in the positive control should be correctly identified, with no target IS detected in the negative controls. |
| **LOD** | + | - | Prepare a series of low-level validation samples by spiking a positive monoclonal cell line (or its DNA) into negative cells (or their DNA). At each level, perform at least three independent experiments, with at least 20 replicate measurements in total. | The LOD is defined as the lowest level at which all known ISs are correctly identified in all replicates. |
| **Robustness** | + | + | Robustness data generated during method development may be used to support validation without additional experiments. Potential sources of variation may include DNA or library storage conditions and duration, equipment models, and reagent brands or lots. Robustness may be evaluated using low-level validation samples in an OFAT study, with at least three replicates under each condition. | For qualitative ISA, deliberate variations in analytical conditions should not affect correct IS detection. For quantitative ISA, results should continue to meet the predefined accuracy and precision criteria. |
| **Accuracy** | - | + | Prepare validation samples at low, medium, and high levels by mixing the test monoclonal cell line (or its DNA) with another positive monoclonal cell line (or its DNA). Analyse three replicates at each level. | Accuracy should be assessed by recovery (measured value/theoretical value ×100%). Recovery should generally be 75%–125%; wider acceptance criteria may be justified for low-level samples, such as those at the LOQ. For monoclonal cell lines harbouring multiple ISs, the contribution of each IS should be considered. |
| **Precision** | - | + | Precision should include evaluation of both repeatability and intermediate precision. Prepare validation samples at low, medium, and high levels by mixing the test monoclonal cell line (or its DNA) with another positive monoclonal cell line (or its DNA), with at least three replicates at each level. Repeatability should be evaluated in a single experiment, whereas intermediate precision should be evaluated in at least three independent experiments. | Precision should be evaluated using the relative standard deviation (RSD). RSD should generally be ≤20%; for low-level samples, such as those at the LOQ, an RSD of ≤25% may be acceptable. For monoclonal cell lines harbouring multiple ISs, precision should be assessed for individual ISs. |
| **LOQ** | - | + | Prepare a series of low-level validation samples by mixing the test monoclonal cell line (or its DNA) with another positive monoclonal cell line (or its DNA). Analyse at least three replicates at each level and evaluate accuracy and precision. | The LOQ is defined as the lowest level at which the predefined accuracy and precision criteria are met. |
| **Linearity and range** | - | + | Prepare validation samples across at least five levels by mixing the test monoclonal cell line (or its DNA) with another positive monoclonal cell line (or its DNA), with at least three replicates per level. | Assess linearity by linear regression of the measured values against the theoretical values. The coefficient of determination (R²) should generally be ≥0.95. The analytical range should comprise levels that meet the predefined criteria for accuracy, precision, and linearity. |

**Supplementary Table S5. Genes Implicated in Integration-Associated Clonal Expansion or Oncogenesis**

| **Gene** | **CGT product type** | **Indication** | **Vector** | **Reported integration-associated effect** | **Clinical consequence** | **References** |
| --- | --- | --- | --- | --- | --- | --- |
| ***LMO2*** | HSPC-GT | SCID-X1; WAS | γ-RV | Insertional activation and overexpression in leukaemic clones | T-cell leukaemia | 1,2 |
| ***CCND2*** | HSPC-GT | SCID-X1 | γ-RV | Insertional activation and overexpression in a leukaemic clone | T-cell leukaemia | 1 |
| ***BMI1*** | HSPC-GT | SCID-X1 | γ-RV | Insertional activation as a potential cooperating event | T-cell leukaemia | 1 |
| ***MECOM* (*MDS1-EVI1*)** | HSPC-GT | X-CGD; WAS; CALD | γ-RV; LV | Insertional activation/dysregulation with myeloid clonal expansion | Clonal expansion; MDS/AML | 2-5 |
| ***PRDM16*** | HSPC-GT | X-CGD; CALD | γ-RV; LV | Insertional activation with myeloid clonal expansion | Clonal expansion; MDS | 3,5 |
| ***SETBP1*** | HSPC-GT | X-CGD | γ-RV | Insertional activation with myeloid clonal expansion | Clonal expansion | 3 |
| ***MN1*** | HSPC-GT | WAS | γ-RV | Integration associated with a dominant leukaemic clone | AML | 2 |
| ***HMGA2*** | HSPC-GT | β-thalassaemia | LV | Aberrant splicing and transcriptional activation in a dominant clone | Clonal dominance without malignant transformation | 6 |
| ***TET2*** | CAR-T | CLL | LV | Gene disruption with marked CAR-T clonal expansion | Clonal expansion without malignant transformation | 7 |
| ***CBL*** | CAR-T | B-ALL | LV | Integration potentially associated with enhanced CAR-T clonal expansion | Clonal expansion without malignant transformation | 8 |
| ***TP53*** | CAR-T | Multiple myeloma | LV | Reduced TP53 expression with a potential contribution to transformation | Secondary CAR-positive T-cell lymphoma | 9 |

AML, acute myeloid leukaemia; B-ALL, B-cell acute lymphoblastic leukaemia; CALD, cerebral adrenoleukodystrophy; CAR-T, chimeric antigen receptor T-cell; CLL, chronic lymphocytic leukaemia; HSPC-GT, haematopoietic stem and progenitor cell gene therapy; MDS, myelodysplastic syndrome; SCID-X1, X-linked severe combined immunodeficiency; WAS, Wiskott–Aldrich syndrome; X-CGD, X-linked chronic granulomatous disease.

Supplementary References

1.	Hacein-Bey-Abina S, Garrigue A, Wang GP, et al. Insertional oncogenesis in 4 patients after retrovirus-mediated gene therapy of SCID-X1. *Journal of Clinical Investigation*. 2008;118(9):3132–3142. doi:10.1172/jci35700

2.	Braun CJ, Boztug K, Paruzynski A, et al. Gene Therapy for Wiskott-Aldrich Syndrome—Long-Term Efficacy and Genotoxicity. *Science Translational Medicine*. 2014;6(227):227ra33. doi:10.1126/scitranslmed.3007280

3.	Ott MG, Schmidt M, Schwarzwaelder K, et al. Correction of X-linked chronic granulomatous disease by gene therapy, augmented by insertional activation of *MDS1-EVI1, PRDM16* or *SETBP1*. *Nat Med*. Apr 2006;12(4):401–9. doi:10.1038/nm1393

4.	Stein S, Ott MG, Schultze-Strasser S, et al. Genomic instability and myelodysplasia with monosomy 7 consequent to *EVI1* activation after gene therapy for chronic granulomatous disease. *Nat Med*. Feb 2010;16(2):198–204. doi:10.1038/nm.2088

5.	Duncan CN, Bledsoe JR, Grzywacz B, et al. Hematologic Cancer after Gene Therapy for Cerebral Adrenoleukodystrophy. *N Engl J Med*. Oct 10 2024;391(14):1287–1301. doi:10.1056/NEJMoa2405541

6.	Cavazzana-Calvo M, Payen E, Negre O, et al. Transfusion independence and *HMGA2* activation after gene therapy of human β-thalassaemia. *Nature*. 2010;467(7313):318–322. doi:10.1038/nature09328

7.	Fraietta JA, Nobles CL, Sammons MA, et al. Disruption of *TET2* promotes the therapeutic efficacy of CD19-targeted T cells. *Nature*. Jun 2018;558(7709):307–312. doi:10.1038/s41586-018-0178-z

8.	Shah NN, Qin H, Yates B, et al. Clonal expansion of CAR T cells harboring lentivector integration in the *CBL* gene following anti-CD22 CAR T-cell therapy. *Blood Adv*. Aug 13 2019;3(15):2317–2322. doi:10.1182/bloodadvances.2019000219

9.	Perica K, Jain N, Scordo M, et al. CD4^+^ T-Cell Lymphoma Harboring a Chimeric Antigen Receptor Integration in *TP53*. *N Engl J Med*. Feb 6 2025;392(6):577–583. doi:10.1056/NEJMoa2411507
